# An Inexpensive Conceptual Training Model for Transvenous Pacemaker Placement

**DOI:** 10.5811/westjem.2019.12.44366

**Published:** 2019-12-19

**Authors:** Timothy P. Young, Jennifer M. Tango, Cory J. Toomasian, Kayla J. Kendric, Deena I. Bengiamin

**Affiliations:** Loma Linda University School of Medicine, Medical Simulation Center, Loma Linda, California. Loma Linda University Medical Center and Children’s Hospital, Department of Emergency Medicine, Loma Linda, California

## Abstract

**Introduction:**

Emergent transvenous (TV) pacemaker placement can be life-saving, but it has associated complications. Emergency medicine (EM) educators must be able to teach this infrequent procedure to trainees.

**Methods:**

We constructed a conceptually-focused, inexpensive training model made from polyvinyl chloride pipes and connectors, vinyl tubing, and a submersible pump. Cost of the model was $51. We tested the model with a group of 15 EM residents. We then asked participants to complete a survey reporting confidence with the procedure before and after the session. Confidence was compared using a Wilcoxon matched-pairs test.

**Results:**

Confidence improved after the session, with a median confidence before the session of 2 (minimally confident; interquartile range [IQR] 1–3) and a median confidence after the session of 4 (very confident; IQR 3–4, p=0.001). All residents agreed that the model helped them to understand the process of placing a TV pacemaker.

**Conclusion:**

Our TV pacemaker placement model was inexpensive and allowed for practice of a complex emergency procedure with direct visualization. It improved trainee confidence.

## BACKGROUND

Emergent transvenous (TV) pacemaker placement is a core emergency medicine (EM) skill.^1^,^2^ While relatively rare, it is potentially life-saving in amenable unstable bradycardia.^3^ However, positioning a temporary TV pacing wire to obtain electrical and mechanical capture is a multi-step, complex task. Complications, including arterial puncture, pneumothorax, myocardial perforation, wound infection, venous thrombosis, line sepsis, diaphragmatic pacing, and pacemaker dislodgement, occurred in 23% of cases where a pacemaker was placed by an EM attending.^4^

EM educators must be able to teach this procedure to trainees. In our training program at a tertiary-care, Level 1 trauma center, residents sometimes do not have the opportunity to perform this procedure on a live patient during their training. We use simulation to address this issue, but have encountered difficulty in demonstrating and practicing the process of TV pacing using standard central line models. In our well-stocked simulation center, we have access to multiple models that allow for placement of blind and ultrasound-guided central lines. However, these models do not simulate the right ventricle or accommodate the length of a TV pacing wire.

Anecdotally, our trainees have commented that the most intimidating factors in TV pacemaker placement are understanding the equipment and remembering the sequence of steps. TV pacemaker placement involves first placing an introducer sheath, which our residents generally have ample opportunities to perform. However, the introducer sheath is generally smaller (6 French) than the more familiar, trauma-sized sheath (8.5 French), and a contamination shield must be correctly loaded onto the wire prior to placement. The process of “floating” the pacemaker using the wire balloon is difficult to conceptualize.

High fidelity has not been shown to have an advantage over low fidelity in simulation.^5^ Simulation models with functional fidelity that lack physical fidelity have shown learning outcomes that are similar to models with both.^6^ We find physical fidelity to be less important in teaching and learning TV pacemaker placement than the ability to demonstrate, practice, and visualize the complete procedure.

## OBJECTIVES

We aimed to build a conceptually-focused model that would allow us to demonstrate emergent TV pacemaker placement inclusive of wire positioning in the right ventricle. We planned to determine the cost of the model and its impact on EM residents’ confidence with the procedure.

## CURRICULAR DESIGN

### Model construction

**Figure 1:**
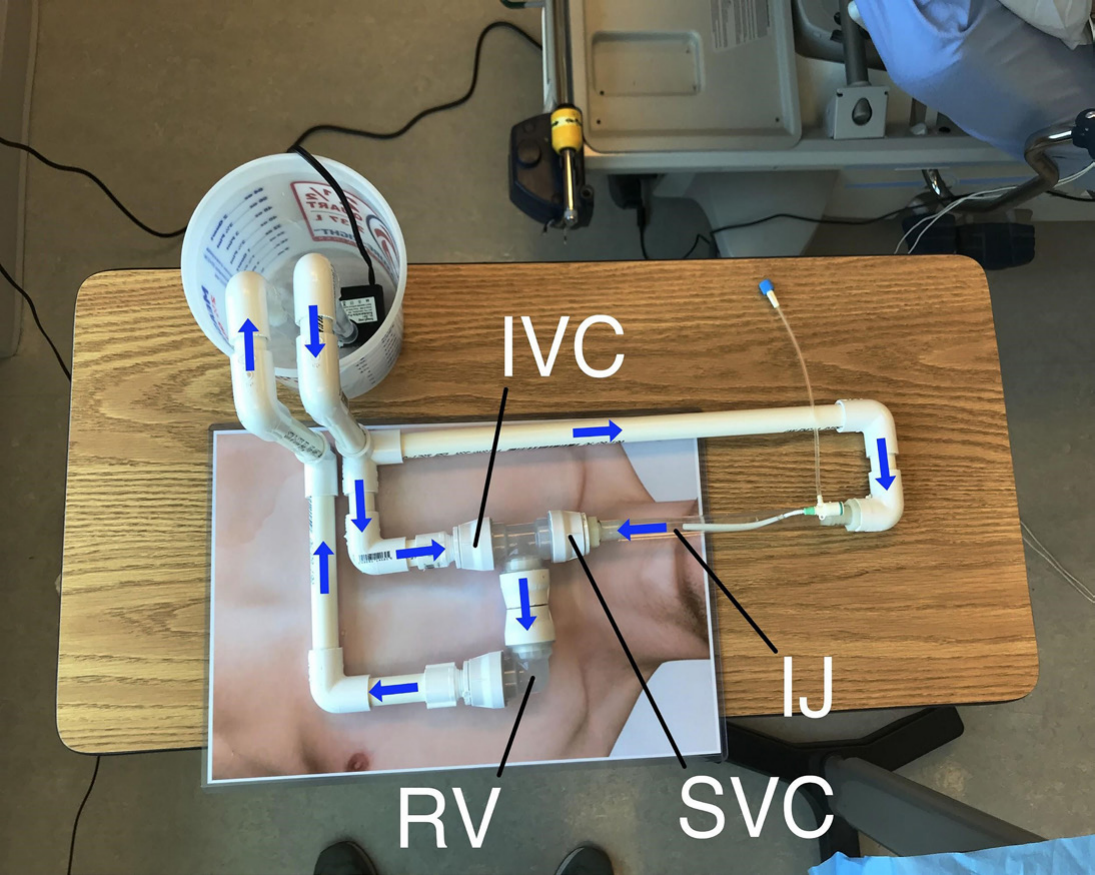
The transvenous pacemaker placement trainer. Arrows denote direction of water flow. *IJ*, internal jugular; *IVC*, inferior vena cava; *RV*, right ventricle; *SVC*, superior vena cava.

We chose to create a functional TV pacemaker placement model instead of a realistic one, using ½-inch opaque polyvinyl chloride (PVC) pipe, clear pipe, vinyl tubing and water. We bought the pipe and tubing locally from a national-chain home improvement store and bought other components online. We used a PVC cutter to cut the pipe to appropriate sizes. We assembled the model to mimic anatomic blood flow, splitting the outflow from the pump to provide directional flow down the superior vena cava (SVC) and up the inferior vena cava (IVC) (Figure 1). This allowed the pacemaker balloon to “float” into the simulated right ventricle in the same manner as it would in a live patient. We drilled a hole in the vinyl tubing and placed a 6 French introducer sheath from our educational equipment supply. To allow air in the system to be expelled, we placed the pump in a 2 ½ quart bucket that acted as a reservoir. Lastly, we printed a photo of a human torso at roughly life-size and placed the model on the photo to allow for learner orientation. Total cost of the model was $51 (Table). It required approximately 2 hours to build.

We created a YouTube video to demonstrate the trainer in use (https://youtu.be/Y1JnOuqtjlg).

**Figure 2:**
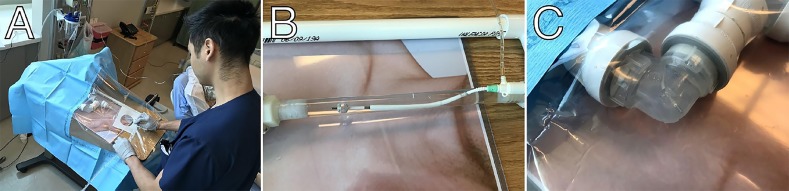
The transvenous pacer trainer in use (A). Inflation of the ballon (B). Wire placed in the right ventricle (C).

We tested the trainer with a group of postgraduate year (PGY)-2 and PGY-3 EM residents (Figure 2). Each resident used the trainer in an individual 30-minute session. At the end of each session, the resident was given a link to a survey and asked to complete it. The survey first asked whether the resident had placed a TV pacemaker in a live patient. The survey then asked the resident to rate confidence in the procedure prior to and after the session on a five-point Likert scale (1=unconfident, 2=minimally confident, 3=confident, 4=very confident, 5=extremely confident). Finally, the resident was asked whether the model helped him or her to understand the process of placing a TV pacemaker.

### Lessons Learned

We found that the model offered several advantages over an opaque model. Two residents inflated the pacing balloon but did not turn the stopcock to trap the air in the balloon. When they removed the syringe, the balloon deflated. The visibility of the model gave them visual feedback and allowed them to correct this mistake and understand why it occurred. This would not have been possible with an opaque trainer or a live patient. The presence of a path to the right ventricle, with the junction between the clear PVC pipes being treated as the tricuspid valve, allowed us to better demonstrate the potential valvular damage that could be done by withdrawing the wire with the balloon inflated. We were able to directly demonstrate why the wire needs to be passed to a minimum distance (such as 20 centimeters [cm]) before inflating the balloon, as residents were able to see the wire exit the sheath at about 13 cm. Preference for the right internal jugular as a site for the introducer sheath was easier to demonstrate with visible vessels. The anatomic, directional flow of water in the SVC and IVC allowed us to explain the function of the balloon.

Initially, the model leaked slowly. This occurred because we tried to connect PVC pieces by friction only. Standard PVC pipe glue at each slip joint resolved this problem. The pacemaker wire sometimes became caught on the lip of the connector, and we needed to redirect it manually by pushing on the outside of the tubing. We found this did not detract from conceptual learning.

## IMPACT/EFFECTIVENESS

Of 15 participants, 13 completed an evaluation (87%). Five residents had placed a TV pacemaker in a live patient. All 13 reported an increase in confidence after the session. We compared confidence levels before and after using a Wilcoxon matched-pairs test. Confidence improved after the session, with a median confidence level before the session of 2 (minimally confident; interquartile range [IQR] 1–3) and a median confidence level after the session of 4 (very confident; IQR 3–4, p=0.001). All residents agreed that the model helped them to understand the process of placing a TV pacemaker.

Our study has limitations. Trainee confidence is considered to be a lower level of training evaluation, and does not guarantee improved behavior or better patient outcomes.^7^ The procedure was placed in isolation, and the relaxed environment may have inflated self-reported confidence. Electrical and mechanical capture are not simulated by our model.

We successfully used the model for one subsequent training session. Maintenance consisted of draining and storing the model between sessions. We plan to implement this model into our regular procedural skills curriculum moving forward. Because of the low cost, we will be able to make the model available for residents longitudinally for just-in-time teaching.

Our TV pacemaker placement model was inexpensive and allowed for demonstration and practice of a complex emergency procedure. It improved trainee confidence in our small sample. Other training programs may find it a good option to teach an important yet difficult-to-simulate procedure.

## Figures and Tables

**Table:** Transvenous pacemaker placement model materials.

Material	Anatomy/function	Price
½ inch PVC pipe and connectors	Vessels	$20
Clear PVC pipe	SVC/IVC/RV	$10
Vinyl tubing	Internal jugular vein	$5
Submersible pump (tinyurl.com/y42u2mn6)	Heart	$10
2 ½ quart plastic bucket (tinyurl.com/yyy3aqqm)	Reservoir	$6
	Total	$51

*PVC*, polyvinylchloride; *SVC*, superior vena cava; *IVC*, inferior vena cava; *RV*, right ventricle.
